# Identifying the risk of dyslexia in bilingual children: The potential of language-dependent and language-independent tasks

**DOI:** 10.3389/fpsyg.2022.935935

**Published:** 2022-11-24

**Authors:** Juhayna Taha, Desire Carioti, Natale Stucchi, Mathilde Chailleux, Elisa Granocchio, Daniela Sarti, Marinella De Salvatore, Maria Teresa Guasti

**Affiliations:** ^1^Department of Psychology, Università degli Studi di Milano-Bicocca, Milan, Italy; ^2^Developmental Neurology Unit, Foundation IRCCS Neurological Institute Carlo Besta, Milan, Italy

**Keywords:** developmental dyslexia, rhythm, bilingualism, RAN, reading, phonological awareness, nonword repetition, sentence repetition

## Abstract

This study investigates the linguistic processing and non-linguistic cognitive abilities of monolingual and bilingual children with and without reading difficulties and examines the relationship between these skills and reading. There were 72 Italian-speaking children: 18 monolingual good readers (MONO-GR, M_age_ = 10;4), 19 monolingual poor readers (MONO-PR, M_age_ = 10;3), 21 bilingual good readers (BI-GR, M_age_ = 10;6), and 16 bilingual poor readers (BI-PR, M_age_ = 10;6). All bilingual children spoke Italian as their L2. Children completed a battery of standardized Italian reading tests, language-dependent tasks: nonword repetition (NWR), sentence repetition (SR), and phonological awareness (PA), and language-independent tasks: timing anticipation, beat synchronization, inhibition control, auditory reaction time, and rapid automatized naming (RAN). Poor readers scored below good readers on the language-dependent tasks, including NWR, PA, and SR. Beat synchronization was the only language-independent task sensitive to reading ability, with poor readers showing greater variability than good readers in tapping to fast rhythms. SR was the only task influenced by language experience as bilinguals underperformed monolinguals on the task. Moreover, there were weak to moderate correlations between performance on some language-dependent tasks (NWR, PA), language-independent tasks (inhibition control, RAN), and reading measures. Performance on the experimental tasks (except for RAN) was not associated with the length of exposure to Italian. The results highlight the potential of NWR, PA, SR, and beat synchronization tasks in identifying the risk of dyslexia in bilingual populations. Future research is needed to validate these findings and to establish the tasks’ diagnostic accuracy.

## Introduction

Up to 10% of school-age children struggle to learn to read due to developmental dyslexia (hereafter dyslexia; Snowling and Melby-Lervåg, 2016; Wagner et al., 2020). The Diagnostic and Statistical Manual of Mental Disorders (DSM-5; American Psychiatric Association, 2013, p. 36–37) defines dyslexia^1^ as a specific learning disorder that impairs reading. Dyslexia is characterized by deficits in accurate and fluent word recognition, poor decoding, and poor spelling abilities. Dyslexia may be associated with additional difficulties in reading comprehension or math reasoning. The long-standing adverse effects of dyslexia are well-documented. Children with dyslexia continue to experience reading problems throughout school-age and beyond, never achieving fluent reading in adolescence (Ferrer et al., 2015) and adulthood (Reis et al., 2020). Moreover, dyslexia is associated with poor educational attainment (McLaughlin et al., 2014), unemployment (Heckman et al., 2013), and a host of behavioral, social, and emotional difficulties (Leitão et al., 2017; Francis et al., 2019). Accurate identification of dyslexia and the provision of timely intervention are therefore necessary to attenuate the adverse consequences associated with the disorder.

Identifying dyslexia in bilingual children is challenging. Here, we define bilingual children as those exposed to a language at home that is different from the majority societal language. Upon school entry, these children learn to read and write in the majority language, i.e., their second language (L2). Converging evidence has shown that bilingual children fall behind monolingual peers in L2 reading comprehension, reading fluency, and spelling (Droop and Verhoeven, 2003; Crosson and Lesaux, 2010; Melby-Lervåg and Lervåg, 2014; Bonifacci and Tobia, 2016). These reading difficulties have been attributed to bilingual children’s weaker L2 vocabulary, grammar, and listening comprehension (Bialystok and Luk, 2012; Melby-Lervåg and Lervåg, 2014; Babayiğit et al., 2022). Essentially, the L2 reading skills of bilingual children are correlated with their L2 oral language skills, which, in turn, are determined by the onset, quantity, and quality of L2 exposure (Paradis et al., 2011). The L2 reading profiles of bilingual children are further influenced by the similarities/differences in the typology/orthography of the languages spoken by the child (Goswami, 2000). Accordingly, bilingual children’s reading and language profiles are highly heterogeneous (Paradis and Grüter, 2014), and reduced exposure to L2 in bilingual children may result in L2 reading profiles similar to those of monolingual children with dyslexia. Therefore, when bilingual children struggle with reading, it is challenging to determine whether these difficulties are due to insufficient L2 exposure or reading impairment. This places bilingual children at a higher risk of being over-identified or under-identified with dyslexia (Deponio et al., 2000; Samson and Lesaux, 2009). Relatedly a recent survey across four European countries, including Italy, revealed a good level of clinicians’ awareness toward bilingual approaches in clinical practice and pointed out the lack of available tools suitable for assessing bilingual children (Bloder et al., 2021).

This paper contributes to the correct identification of dyslexia in linguistically diverse children. Specifically, we examine two promising approaches to assessing dyslexia in monolingual and bilingual Italian-speaking children. The first approach considers language-dependent tasks that tap into language processing abilities, including phonological awareness, nonword repetition, and sentence repetition. The second approach considers language-independent tasks that tap into non-linguistic cognitive skills, including rhythmic timing, inhibition control, and naming speed. Extensive literature shows that linguistic processing and non-linguistic cognitive abilities are compromised in monolingual children with dyslexia, suggesting that these skills are closely correlated with reading development. Linguistic processing and non-linguistic cognitive skills appear to share underlying cognitive mechanisms with reading and could be sensitive to reading difficulties. Language-dependent processing tasks emphasize the processing rather than knowledge of linguistic material, whereas language-independent tasks involve minimal linguistic content. Hence, both types of tasks may be less affected by the level of proficiency in a given language (Ebert and Pham, 2019), making them suitable for assessing children with diverse linguistic backgrounds.

In this study, we systematically investigate the impact of bilingualism and reading ability on the performance on a set of language-dependent and language-independent tasks. This is to characterize similarities and differences in monolingual and bilingual children’s linguistic and cognitive skills and investigate whether impairments in these abilities can differentiate children with and without reading difficulties. In the following sections, we review the research evidence on linguistic and non-linguistic cognitive deficits in children with dyslexia and discuss how these abilities relate to reading.

### Linguistic processing deficits of children with dyslexia

The etiology of dyslexia is debated (Peterson and Pennington, 2015; Snowling et al., 2020). A widely held view attributes dyslexia to a deficit in the representation, encoding, storage, access, and processing of phonological information. This deficit is thought to interfere with learning the grapheme-phoneme correspondences that support letter-to-sound decoding and word recognition (Stanovich, 1988; Stanovich and Siegel, 1994; Snowling, 1998; Lyon et al., 2003; Ramus, 2003, 2014). A large body of research shows that children with dyslexia have impairments in phonological processing (for a review, see Vellutino et al., 2004; Peterson and Pennington, 2015; Stein, 2018).

The deficits in **phonological awareness,** i.e., the conscious ability to detect and manipulate speech segments of a language (e.g., syllables, rhymes, and phonemes; Scarborough and Brady, 2002), are regarded as a robust cross-linguistic marker of dyslexia (Goswami, 2000; Ramus, 2003; Peterson and Pennington, 2015). School-age children with dyslexia underperform age-matched typically developing (TD) on a range of phonological awareness measures such as spoonerism, letter knowledge, phonemic awareness, rhyme production and discrimination, and syllable deletion (for a meta-analysis, see Kudo et al., 2015). Importantly, robust literature suggests that children with dyslexia do not acquire appropriate phonemic awareness levels irrespective of age or reading level (Bruck, 1992). For instance, Melby-Lervåg et al. (2012b) reported that, relative to age-matched and reading level-matched TD children, children with dyslexia demonstrate a significant, profound deficit in phonemic awareness, including phoneme deletion, segmentation, blending detection, and spoonerism tasks. This pattern has been replicated in transparent orthographies such as Italian (Tobia and Marzocchi, 2014; Fastame et al., 2018; Parrila et al., 2020; Vender and Melloni, 2021). Across orthographies, studies revealed that phonemic awareness has a fundamental role in early reading development (Bradley and Bryant, 1983; Snowling et al., 2003; Ziegler and Goswami, 2005; Melby-Lervåg et al., 2012b; Carroll et al., 2014).

Bilingual children may have superior phonological awareness skills relative to monolingual peers. This bilingual advantage is documented in bilingual children who speak pairs of languages varying in phonological complexity and orthographic depth and across different tasks (Bruck and Genesee, 1995; Campbell and Sais, 1995; Bialystok et al., 2003; Marinova-Todd et al., 2010; Kang, 2012). By the end of first grade, bilinguals and monolinguals tend to have comparable phonological awareness skills (Bruck and Genesee, 1995; Campbell and Sais, 1995; Bialystok et al., 2003; Pawlicka et al., 2018; Vender and Melloni, 2021).

Several studies have examined phonological awareness skills of bilingual children with dyslexia (Frederickson and Frith, 1998; Chiappe and Siegel, 1999; Everatt et al., 2000; Chung and Ho, 2010; Ijalba et al., 2020; Vender and Melloni, 2021). Chiappe and Siegel (1999) compared the phonological awareness skills of monolingual English-speaking children and bilingual Punjabi-English-speaking children in first grade. The authors found that phonological awareness successfully discriminated between average and poor readers but did not discriminate between monolingual and bilingual children. Vender and Melloni (2021) assessed phonological awareness performance in 10-year-old, Italian-speaking children with and without dyslexia. Bilingual children with and without dyslexia performed similarly to their monolingual counterparts in nonword repetition, rhyme detection, and spoonerism tasks. Notably, dyslexic monolingual and bilingual children performed below TD children in the three tasks. The evidence indicates that the performance of school-age children on L2 phonological awareness tasks is diminished by dyslexia but is not influenced by bilingual experiences. Hence, poor phonological awareness could be a valuable indicator of dyslexia in children with diverse linguistic backgrounds.

Verbal short-term memory, traditionally assessed using **nonword repetition (NWR)** and serial order recall tasks, is also compromised in children with dyslexia (for reviews, see Melby-Lervåg and Lervåg, 2012; Majerus and Cowan, 2016). In NWR tasks, children are asked to repeat unfamiliar phonological forms that conform to the phonotactics of a language yet lack any meaning. Besides verbal short-term memory, NWR involves auditory perception, encoding of phonological information, motor planning, and articulation (Coady and Evans, 2008; Archibald et al., 2013; Pigdon et al., 2019). NWR performance is associated with individual differences in language and reading development (Bowey, 1997; Gray, 2003; Gathercole, 2006; Adlof and Patten, 2017; Vender et al., 2020; Cunningham et al., 2021). Numerous studies have indicated that children with reading and language difficulties score below TD peers in NWR tasks (see Graf Estes et al., 2007; Melby-Lervåg and Lervåg, 2012; Ehrhorn et al., 2020; Schwob et al., 2021). Monolingual children with dyslexia reportedly perform poorly on NWR compared to chronological age- and reading level-matched TD children (Melby-Lervåg and Lervåg, 2012). These difficulties have been documented across many languages (Suk-Han Ho and Ngar-Chi Lai, 1999; Boets et al., 2010; Vender et al., 2020).

Bilingual TD children may underperform monolingual peers in L2 NWR tasks (Kohnert et al., 2006; Engel de Abreu, 2011; Boerma et al., 2015). Interestingly, research on Italian reported comparable performance among monolingual and bilingual preschool and school-age TD children on Italian NWR tasks (Guasti et al., 2013; Vender et al., 2016, 2020; Vender and Melloni, 2021). The discrepancy in results between studies may be attributed to methodological factors such as bilingual sample characteristics and L2 exposure patterns. Vender et al. (2016) further explain that Italian phonology has simpler syllabic forms, a lower number of consonant clusters, and a smaller phonemic inventory. Accordingly, NWR appears to be less demanding for bilingual children whose L2 has simple phonotactic structures, like Italian (Vender et al., 2016; Melloni and Vender, 2020).

Vender et al. (2020) found that Italian-speaking, monolingual and bilingual children with dyslexia scored below their monolingual peers on an Italian nonword repetition task. Conversely, no NWR differences were found between monolingual and bilingual children. Accordingly, while L2 NWR performance is compromised by dyslexia, it is not influenced by bilingualism, at least when L2 has a simple phonotactic structure, like Italian (Vender et al., 2020). Consistent with these findings, performance on the Italian NWR task correlated with Italian reading proficiency but not Italian vocabulary knowledge or Italian exposure indices (e.g., age of first exposure, quantity, and length of exposure to Italian). Performance on the NWR task correctly identified 83% of children in the dyslexic groups and 85% of children in the TD groups (Vender et al., 2020). Overall. NWR may be a non-biased linguistic measure for identifying dyslexia in bilingual children acquiring Italian as their L2.

Less research has examined language abilities outside the domain of phonology in children with dyslexia. Children with or at risk of dyslexia often exhibit a history of early language delay during preschool years (see Vellutino et al., 2004; Snowling and Melby-Lervåg, 2016). These difficulties may persist into the school years, and many children with dyslexia are later identified as having developmental language disorder (DLD; McArthur et al., 2000; Catts et al., 2005; Adlof and Hogan, 2018; Snowling et al., 2020). Adlof and Hogan (2018) emphasize that children with dyslexia, with or without co-morbid DLD, may exhibit broader oral language deficits. Children with dyslexia have morpho-syntactic difficulties, as evidenced by their lower performance on standardized tests of grammar (McArthur et al., 2000), production and comprehension of syntactic structures (Leikin and Assayag-Bouskila, 2004; Robertson and Joanisse, 2010; Talli et al., 2013; Guasti et al., 2016; Arosio et al., 2017; Delage and Durrleman, 2018), verbal morphology (Joanisse et al., 2000; Cantiani et al., 2013; Van Witteloostuijn et al., 2021), and **sentence repetition** (SR; Plaza et al., 2002; Ramus et al., 2013; Moll et al., 2015; Dosi and Koutsipetsidou, 2019). SR can capture the language difficulties in monolingual children with dyslexia (Moll et al., 2015) and children with a history of language difficulty in whom overt symptoms have resolved (Hesketh and Conti-Ramsden, 2013).

Some studies showed that bilingual TD children might score below monolingual children on L2 SR tasks. This pattern was observed in Farsi-English (Komeili and Marshall, 2013), Turkish-English (Chiat et al., 2013), and Russian-Hebrew (Armon-Lotem and Meir, 2016). It has been reported that bilingual children’s performance on SR correlated with the amount of language exposure in L2, such as length of exposure (LoE; Komeili and Marshall, 2013; Thordardottir and Brandeker, 2013; Armon-Lotem and Meir, 2016; Pratt et al., 2021). However, SR performance is not always sensitive to LoE (Chiat et al., 2013; Meir et al., 2016). Meir et al. (2016) suggested that SR can be used with children after 12 months of exposure to L2. To our knowledge, no studies have examined SR in bilingual children with dyslexia. Yet, the available literature indicates that bilingual children with DLD perform below their TD peers on SR and that the task can reliably discriminate between bilingual children with and without DLD (Thordardottir and Brandeker, 2013; Armon-Lotem and Meir, 2016; Meir et al., 2016; Tuller et al., 2018; Pratt et al., 2021). These studies show that SR is an area of vulnerability in children with dyslexia and is a universal clinical marker of DLD (Rujas et al., 2021), which often co-occurs with dyslexia. This evidence sheds light on the potential contribution of SR to increasing the accuracy of dyslexia diagnosis in linguistically diverse children.

### Non-linguistic cognitive deficits in children with dyslexia

Children with dyslexia exhibit several non-linguistic deficits, including impairments in temporal processing, cerebellar and magnocellular functioning, procedural learning, visual processing, and attention (for reviews of non-linguistic theories, see Wright et al., 2000; Ramus, 2003; Laasonen et al., 2020). In this study, we explore the impairments in the perception and production of rhythmic patterns (for a review, see Ladányi et al., 2020). Experimental studies have found that children with dyslexia exhibit inefficient processing of auditory cues to rhythmic timing of speech, particularly amplitude envelope onset, i.e., rise time (for a summary, see Goswami, 2011, 2015). Children with dyslexia also experience difficulties with musical timing skills (Overy et al., 2003; Huss et al., 2011; Caccia and Lorusso, 2021). Overy et al. (2003) demonstrated that children with dyslexia perform below TD children in musical timing tasks involving rhythmic skills (e.g., same/different rhythm discrimination, tapping the rhythm of a song), meter skills (e.g., fast/slow tempo discrimination), rapid skills (e.g., determining the number/order of rapidly presented notes). Huss et al. (2011) found that children with dyslexia were poorer than age-matched TD children in discriminating metrical structures in musical sequences.

The perception of auditory rhythmic patterns in speech and music correlates with individual differences in reading. Time rise sensitivity is related to variations in phonological awareness (e.g., rhyme awareness and phoneme segmentation), reading and spelling skills in children with and without dyslexia (Goswami et al., 2002; Muneaux et al., 2004; Richardson et al., 2004; Thomson and Goswami, 2008; Surányi et al., 2009; Huss et al., 2011). In children with and without dyslexia, metrical perception ability predicted phonological awareness, reading, and spelling even when age, IQ, and short-term memory were controlled (Huss et al., 2011). Anvari et al. (2002) found that musical pitch processing (same/different melody and chord discrimination) positively correlated with phonemic awareness and reading. In children with dyslexia, Flaugnacco et al. (2014) showed that alongside IQ, meter perception abilities predicted text reading accuracy and word reading speed, whereas rhythm reproduction predicted nonword reading accuracy. Moreover, individual differences in beat perception in children with and without dyslexia predicted nonword reading, RAN, and phonological awareness (rhyme oddity; Muneaux et al., 2004). Ozernov-Palchik and Patel (2018) reported that rhythm discrimination predicted letter-sound knowledge even after controlling for IQ and nonword repetition. Longitudinally, rhythm reproduction in kindergarten predicted second-grade reading ability even when short-term memory, attention, and processing speed were partialled out (Dellatolas et al., 2009). Similarly, Moritz et al. (2013) documented that rhythm production and rhythm discrimination skills in kindergarten predicted second-grade phonological and reading performance. These findings imply that rhythm perception plays a role in reading acquisition. Rhythm perception requires the perception of the temporal structure of sound. This skill is also necessary for speech perception. Hence, imprecise perception of auditory timing cues may result in less-specified phonological representations, leading to delays in phonological awareness and adversely affecting reading development (Goswami et al., 2002, 2010; Huss et al., 2011; Ozernov-Palchik and Patel, 2018).

According to the inefficient anticipation hypothesis (Guasti et al., 2017; Pagliarini et al., 2020; Taha et al., 2022), rhythm is defined as a pattern of successive events. Rhythm is viewed as a basic periodic pulsation plus an organization of temporal events that creates a time gestalt. Accordingly, two skills underly rhythmic behaviors: synchronization and structural anticipation.

**The first component of rhythm, i.e., synchronization,** refers to the ability to coordinate one’s motor movement with a repetitive periodic pulsation or a metronome (also known as beat synchronization). This skill appears to be somewhat compromised in children with dyslexia (Wolff, 2002; Overy et al., 2003; Thomson and Goswami, 2008; Waber et al., 2010; Colling et al., 2017). Thomson and Goswami (2008) examined rhythmic finger tapping in paced (tapping to pacing metronome beats) and unpaced conditions (continuous tapping after metronome beats stop). Children with and without dyslexia were asked to tap in time to three metronome rates (1.5, 2, and 2.5 Hz). Relative to TD children, the tapping rates of children with dyslexia deviated more from the expected tapping rates of the paced 2 and 2.5 Hz conditions.

On the other hand, there were no group differences in any tapping rates of the unpaced condition. Additionally, children with dyslexia showed more significant variation in their tapping rates at 2 and 2.5 Hz of the paced condition and in tapping at 2.5 Hz of the unpaced condition. These results indicate that children with dyslexia synchronize less precisely to isochronous rhythms presented in the auditory domain than TD children. Impairments in beat synchronization are also associated with DLD, a condition that often co-occurs with dyslexia (Corriveau and Goswami, 2009).

Relatedly, several studies have reported associations between beat synchronization skills, phonological processing of language, and literacy acquisition. Thomson and Goswami (2008) noted that children who were less consistent in maintaining the target tapping rate were those who had poorer reading, spelling, and phonological awareness development. Furthermore, tapping consistency predicted reading, spelling, and phonological awareness (rhyme oddity) even when age, verbal and non-verbal IQ, and auditory processing (rise time discrimination) were controlled. This suggested that the contribution of rhythmic motor timing skills to literacy development was independent of the ability to perceive auditory rhythmic cues in speech (Thomson and Goswami, 2008). Similarly, in a combined sample of children with and without DLD, Corriveau and Goswami (2009) found that paced tapping accuracy predicted vocabulary, phonological awareness (phoneme deletion, rime awareness), nonword repetition, RAN, word and nonword reading, spelling, and reading comprehension. Flaugnacco et al. (2014) reported that dyslexic children paced tapping ability correlated with word reading accuracy, word reading speed, nonword repetition, and phoneme blending. Even before exposure to reading, 3- to 4-year-old TD children with better beat synchronization skills also have better prereading language skills, including phonological awareness, verbal short-term memory (sentence recall), and naming speed (Kali Woodruff et al., 2014). Yet, not all studies have found these correlations. Lundetræ and Thomson (2018) found that beat synchronization ability at school entry (alongside short-term memory, letter knowledge, initial phoneme isolation, and family risk for reading/writing difficulties) predicted spelling performance but not reading at the end of first grade.

**The second component of rhythm, i.e., anticipation,** refers to the ability to utilize the structural regularity of a rhythmic pattern to orient oneself in time, create a representation of future events in advance, and be prepared to act. Anticipation is also impaired in children with dyslexia. In a recent study, Pagliarini et al. (2020) asked Italian-speaking adults and children with and without dyslexia to perform a tapping task following the warning-imperative paradigm (Walter et al., 1964). In the familiarization phase, participants listened to a regular rhythmic sequence, which allowed the generation of an abstract temporal representation of the heard sequence. In the testing phase, they were presented with the same rhythmic sequence, which additionally contained couples of randomly distributed adjacent tones: a warning beat (WI), which alerted participants (i.e., put them in anticipatory mode) and cued the arrival of the second imperative beat (IB), to which participants had to tap to. The TD children, on average, tapped 40 milliseconds before the IB, suggesting that they anticipated it.

On the other hand, children with dyslexia, on average, tapped 46 milliseconds after the IB, indicating delayed anticipation. Moreover, children with dyslexia were generally less consistent than TD children in tapping to the IB. Pagliarini et al. (2020) reported that children who were less consistent and had more significant timing errors in anticipating the IB also showed slower reading speed (words and nonwords) and made more reading errors. These relationships were also evident in the adult data even when motor dexterity was controlled. In another study, Pagliarini et al. (2015) showed that children with dyslexia fail to comply with the rhythmic constraints of handwriting, and this difficulty is proportionate to reading and language abilities.

The inefficient anticipation hypothesis postulates that anticipation is fundamental in phenomena that unfold in time, particularly those which require precise timing and sequential ordering of behavioral patterns. Examples of such phenomena include language, music, handwriting, and reading. Hence, the inefficient anticipation mechanisms in children with dyslexia may explain the observed impairments in beat synchronization, reading, handwriting, and predictive language processing (Taha et al., 2022; Guasti et al., 2017; Pagliarini et al., 2020).

Executive functioning, particularly inhibition control, has an essential role in anticipation. Children with dyslexia show impairments in inhibition control (Reiter et al., 2005; Doyle et al., 2018; Barbosa et al., 2019). Some studies report higher levels of inhibition control in bilinguals than in monolinguals, whereas other studies document comparable performance (Arizmendi et al., 2018). In one study on Italian-speaking children, Bonifacci et al. (2011) did not find significant differences between bilinguals and monolinguals in inhibition control (as assessed by the Go/No-Go task). In a recent study, Arizmendi et al. (2018) found no significant differences between 7- to 9-year-old monolingual and bilingual children in inhibition control tasks. The results indicate that inhibition control is minimally influenced by language experience and may be valuable for diagnosing dyslexia in bilingual children.

According to the inefficient anticipation hypothesis, children with dyslexia cannot exploit upcoming parafoveal information during reading (De Luca et al., 2013) or **rapid automatized naming** (**RAN;** Pan et al., 2013), resulting in slower performance in these two tasks. RAN refers to the ability to rapidly and accurately name a series of visually presented symbols such as digits, letters, or objects (Denckla and Rudel, 1976). There is a debate about what RAN precisely measures. In this study, RAN is considered a measure of automaticity that taps into several integrative processes, such as phonological processing, visual-lexical access, visual-attention processing, motor planning, and articulation (Wolf and Bowers, 1999; Cummine et al., 2015). Wolf and Bowers (1999) have proposed that slow RAN is the second hallmark of dyslexia. Children with dyslexia require more time and make more errors in RAN relative to same-age TD peers, irrespective of the orthographic consistency of one’s language (for reviews, see Araújo and Faísca, 2019; Parrila et al., 2020; Carioti et al., 2021). Empirical investigations have established RAN as a predictor of reading ability, both concurrently and longitudinally (Adlof et al., 2010; Mazzocco and Grimm, 2013). This relationship is evident across languages with varying levels of orthographic depths (e.g., Caravolas et al., 2013; Georgiou et al., 2016; Landerl et al., 2018; Carioti et al., 2021). Given the link between reading and RAN and the observed persisting deficits in RAN in individuals with dyslexia, researchers have recommended RAN as a long-term, cross-linguistic marker of dyslexia (Araújo et al., 2015; Araújo and Faísca, 2019; Carioti et al., 2022a).

There is a shortage of research on RAN tasks’ usefulness for identifying reading difficulties in bilingual children. Recently, Carioti et al. (2022a) showed that RAN of shapes (the same version used in the present study) could identify reading difficulties in both Italian-speaking monolingual and bilingual language minority children acquiring Italian as their L2. RAN could be useful for identifying reading difficulties in children regardless of their linguistic experiences.

### Study aims, questions, and predictions

The assessment of L2 reading abilities in bilingual children is complicated by many factors related to L2 exposure and proficiency. The high variability in the L2 reading profiles of bilingual children makes it difficult for educators and clinicians to determine whether the L2 reading difficulties in these children reflect a distinct stage of L2 reading acquisition or an underlying reading impairment. This challenge highlights the need for assessment tools that are less influenced by a child’s language experiences. This study addresses these issues by considering the potential of language-dependent tasks (PA, NWR, and SR) and language-independent cognitive tasks (timing anticipation, beat synchronization, inhibition control, and RAN). On the one hand, there is an overlap in the foundations of reading acquisition, language processing, and cognitive abilities, and developmental inefficiencies in these skills are expected to hinder reading development. Therefore, they are sensitive to dyslexia. On the other hand, these tasks either rely on manipulation rather than knowledge of linguistic material or involve minimal or no linguistic stimuli, thus minimizing the effect of L2 proficiency on performance. These features make such tasks valuable for improving diagnosing dyslexia in bilingual children. We aim to address the following research questions.

#### Does performance on language-dependent and language-independent tasks differ by reading ability and/or bilingualism status, and if so, do these two factors interact?

In regards to the language-dependent tasks, consistent with previous Italian studies (Vender et al., 2016, 2020; Vender and Melloni, 2021), we anticipate that performance on the PA and NWR will be sensitive to reading ability, i.e., poor readers will perform below good readers but will not be influenced by bilingualism status. Children with dyslexia have weaker language skills (McArthur et al., 2000; Moll et al., 2015; Adlof and Hogan, 2018). Therefore, we predict that performance on SR would differ by reading ability, where poor readers will underperform good readers. Given the conflicting evidence on the performance of bilinguals on SR, bilingualism may or may not impact performance on the task.

In terms of the language-independent tasks, based on previous findings (Thomson and Goswami, 2008; Pagliarini et al., 2020), we anticipate that performance on timing anticipation and beat synchronization tasks will differ by reading ability. Poor readers are expected to show impairments in these tasks relative to good readers. Because these tasks involve minimal linguistic content, the effect of bilingualism on performance is expected to be minimal. Inhibition control impairments have been reported in children with dyslexia (Reiter et al., 2005; Doyle et al., 2018). The bilingual advantage in school-aged Italian-speaking children is not robust in inhibition control tasks (Bonifacci et al., 2011). Based on this evidence, it is predicted that inhibition control will be compromised by reading difficulties but will not be influenced by bilingualism status. Consistent with Carioti et al. (2022a), poor readers, both monolinguals and bilinguals, are expected to be slower than good readers in RAN of shapes. We do not predict to find a bilingualism effect on RAN.

#### How do language processing, language-independent cognitive and rhythmic abilities, and reading correlate in monolingual and bilingual children?

Given the role of phonological abilities in reading acquisition (Snowling, 1998; Ramus et al., 2013) and sentence repetition in learning (Alloway and Gathercole, 2005; Moll et al., 2015), we predict positive correlations between PA, NWR, and SR with reading performance.

Based on prior evidence, we hypothesize that anticipation (Pagliarini et al., 2020), beat synchronization (Thomson and Goswami, 2008; Corriveau and Goswami, 2009), inhibition control (Bonifacci et al., 2011; Kieffer et al., 2013), and RAN will be associated with reading as well as language abilities. We further hypothesize that performance on Italian PA and NWR tasks will not be related to factors of Italian language exposure (Vender et al., 2016, 2020). On the other hand, performance on SR may rely more on linguistic knowledge and may therefore be associated with measures of Italian language exposure (Thordardottir and Brandeker, 2013; Armon-Lotem and Meir, 2016; Pratt et al., 2021).

Lastly, timing anticipation, beat synchronization, and inhibition control tasks do not involve linguistic material. Hence, we predict that performance on these tasks will be independent of Italian language exposure. The RAN task administered here does not require extensive knowledge of Italian linguistic structures. Instead, it only requires basic lexical knowledge of five shapes only. Accordingly, RAN is not entirely language-independent. We, therefore, cannot exclude that RAN may correlate with other linguistic measures (e.g., SR) and/or measures of Italian language exposure. Assuming that RAN taps automation skills and processing speed, we predict that RAN will correlate more strongly with reading speed than reading accuracy.

## Materials and methods

### Participants

This study received approval from the Research Ethics Committees at the University of Milano-Bicocca (n.461; 7/06/2019) and the Foundation IRCCS Neurological Institute Carlo Besta (n.02; 2/4/2022). Parents or guardians provided written informed consent for their children to participate in the study, and all children gave verbal assent.

Data were initially collected from 104 children residing in the province of Milan, Italy. The general eligibility criteria were: a chronological age between 9 and 12 years, having Italian as the first or second language, and no known diagnosis of cognitive, sensory, neurological, or motor impairments. To confirm within-normal nonverbal intelligence, children up to the age of 11;6 years were required to score at or above the 16^th^ percentile on Raven’s colored progressive matrices (Raven et al., 1998; Italian adaptation; internal consistency =0.91; Belacchi et al., 2008); children aged 11;7 years or older had to obtain an IQ score of 85 or above on the non-verbal subset of the Wechsler Intelligence Scale for Children-IV (Wechsler, 2003, Italian standardization; test–retest reliability =0.92; by Orsini et al., 2012).

Seventy-four children aged between 9;0 and 11;10 years (38 females, 35 males, M_age_ = 10;4, SD = 0;10) met the inclusionary criteria described above and were selected for this study. To cover the spectrum of reading abilities, children were recruited from two sources: primary and elementary classrooms of a mainstream school and the Developmental Neurology Unit of Foundation IRCCS Neurological Institute Carlo Besta in Milan.

#### Determining the monolingual and bilingual status of children

Information about the linguistic backgrounds of the children was collected *via* the *Parents of Bilingual Children Questionnaire* (PaBiQ, Tuller, 2015). The fourth author adapted a shortened version of PaBiQ that included 23 questions across six sections: (1) the child’s early language development, including the age of first word and the age of first sentence, (2) early parental concerns, (3) family history of language and/or literacy difficulties (4) the age and length of contact with the different languages, (5) current amount and diversity of contexts of language input, and (6) parent’s education, occupation, and language proficiency. Children classified as monolinguals were those who used Italian at home and school and had minimal or no exposure to other languages, as indicated by PaBiQ. Children classified as bilinguals spoke one or more languages besides Italian and used the language(s) at home with at least one household member. The home languages spoken by the bilingual children were diverse and included: Spanish, Arabic, Chinese, Filipino, Albanian, Romanian, Portuguese and Ukrainian.

Multiple measures were derived from the PaBiQ. The **Positive Early Development Index (/14 points)** was based on the timing of early language milestones and parental concerns and was calculated as the sum of (1) and (2). The **Family History Index** was based on (3) reflecting risk factors for language and literacy disorders. The **No Risk Index (/23 points)** was synthesized as the sum of the Positive Early Development Index and Positive Family History Index. Additional scores were calculated for the bilingual children, such as the **age of onset (AoO)** of consistent exposure to Italian (listening and speaking), the **Length of Exposure (LoE)** to Italian, the **current use of Italian at home (/16 points)** which reflected the frequency of use of Italian between the child and immediate family members at home, and the **Italian linguistic richness (/14 points)** which indicated the frequency of use of Italian during extracurricular activities and with friends (see Table 1; for details of scoring procedures, see Tuller, 2015). All bilingual children had a minimum of 2 years of exposure to Italian, ensuring sufficient knowledge to complete the language and reading assessments.

**Table 1:** Participants’ demographic characteristics, language background, and performance on the standardized reading assessments.

	Group				
Variable	Mono-GR *N* = 18	Mono-PR *N* = 19	Bi-GR *N* = 21	Bi-PR *N* = 16	Group difference
**Demographics**					
Age (in years)	10;4 (0;10)	10;3 (0;10)	10;6 (0;9)	10;6 (0;11)	NO
Gender	11 M, 7 F	11 M, 8 F	14 M, 7\u00B0F	6 M, 10\u00B0F	NO
Non-verbal IQ (Raven’s percentile)	61.39 (21.14)	58.74 (31.68)	56.52 (28.13)	53.36 (28.13)	NO
**PABIQ measures** ^*^					
Positive early development (/14)	12.53 (3.25)	12.11 (3.02)	12.50 (2.04)	11 (3.40)	NO
Family risk (/9)	8.71(0.78)	7.53 (1.47)	8.10 (1.38)	7.86 (1.51)	PR < GR
No risk index (/23)	19.89 (5.06)	19.63 (3.53)	20.65 (2.68)	18.50 (3.40)	NO
AoO Italian—listening (months)			14.33 (27.08)	23.64 (35.37)	NO
AoO Italian—speaking (months)			32.29 (23)	40.36(25.92)	NO
LoE Italian (months)			111.25(27.80)	102.50(32.11)	NO
Use of Italian at home (/16)			8.40(3.15)	7.67(3.68)	NO
Italian language richness (/14)			8.10(1.55)	8.27(1.58)	NO
**Reading measures** ^*^					
Word reading fluency	0.20(0.96)	−1.92(0.64)	−0.22(0.84)	−1.48(0.80)	PR < GR
Word reading accuracy	−0.13(0.75)	−1.87(1.55)	−0.54(1.08)	−2.54(1.40)	PR < GR
Nonword reading speed	0.30(0.98)	−1.41(0.87)	0.06(0.62)	−0.91(0.67)	PR < GR
Nonword reading accuracy	−0.12(0.91)	−1.38(1.26)	0.16(0.65)	−1.27(1.15)	PR < GR
Text reading speed	−0.23(0.88)	−2.37(0.74)	−0.018(0.84)	−2.07(0.97)	PR < GR
Text reading accuracy	−0.77(1.39)	−2.76(1.56)	−0.52(0.84)	−3.54(3.83)	PR < GR

The numbers are reported as Mean (SD). Mono-GR, monolingual good readers; Mono-PR, monolingual poor readers; Bi-GR, bilingual good readers; Bi-PR, bilingual poor readers; M, male; F, female; PABIQ, Parents of Bilingual Children Questionnaire (Tuller, 2015); AoO, age of onset; LoE, length of exposure. For all reading measures, standardized scores are reported.

* Descriptive statistics, analyses for PaBIQ and reading measures are reported after removal of outliers.

#### Determining the reading ability

The children’s reading ability was determined based on their performance on a set of standardized reading assessments. Single word and nonword reading speed and accuracy were assessed using the 2nd and 3rd subsets of the Battery for the Assessment of Italian Developmental Dyslexia and Dysorthography—second edition (DDE-2, Sartori et al., 2007). The test–retest reliability for this battery (as assessed by correlation coefficients) is 0.77 for speed and 0.56 for accuracy, while the concurrent validity ranges from 0.74 to 0.96 (Sartori et al., 2007). Text reading speed and accuracy were evaluated using the Reading and Comprehension Assessment for Elementary and Middle School (PROVE-MT-3-Clinica, Cornoldi and Carretti, 2016). The test–retest reliability for the texts of this battery for all grades is 0.85 (Cornoldi et al., 2017).

Poor reading ability was defined as a score at or below −1.5 SD of the normative mean on at least two reading measures, and good reading ability was described as a score above −1.5 SD of the normative mean on five of the reading measures. It is important to note that the criteria employed in this study are not as restrictive as the ones suggested by the Italian diagnostic guidelines (e.g., scoring −2 SD below the mean on two or more reading measures). Therefore, in the present study, “poor reading” refers to students who struggle with reading. Some, but not all, of these poor readers have a dyslexia diagnosis.

#### The final sample of participants

The final sample comprised four groups: 18 monolingual good readers (MONO-GR), 19 monolingual poor readers (MONO-PR), 21 bilingual good readers (BI-GR), and 16 bilingual poor readers (BI-PR). There were 29 children (17 MONO-PR and 12 BI-PR) with an official diagnosis of dyslexia according to the (DSM-5) guidelines. Of these children, eight were additionally diagnosed with DLD. Table 1 summarizes the participants’ characteristics. There were no significant differences in chronological age between the monolingual (MONO) and bilingual (BI) groups, *F*(1,70) =0.99, *p* = 0.324 or between the good readers (GR) and poor readers (PR), *F*(1,70) =0.12, *p* = 0.726. There were no significant differences in non-verbal abilities (as indexed by Raven’s scores) between the MONO and BI children, *F*(1,68) =0.66, *p* = 0.420 or between the GR and PR groups, *F*(1,67) =0.14, *p* = 0.713. Furthermore, socio-economic status, as indexed by the maternal education level, did not differ significantly across the groups (*Fisher’s exact test*-*p* = 0.434).

Parental responses on the PaBiQ revealed that the Positive Early Development Index scores did not differ significantly among the MONO and BI groups, χ^2^(1) = 0.92, *p* = 0.339, nor between the GR and PR groups, χ^2^(1) = 0.59, *p* = 0.443. The Family Risk scores were comparable between the BI and MONO groups, χ^2^(1) = 0.11, *p* = 0.741, but differed significantly between the PR (*M* = 7.67, *SD* = 1.47) and the GR groups (*M* = 8.37, *SD* = 1.17; χ^2^(1) = 6, *p* < 0.05). The No Risk Index scores were comparable among the MONO and BI-groups, χ^2^(1) =0.18, *p* = 0.672 and between the GR and PR groups (χ^2^(1) = 3.88, *p* = 0.05).

A Mann–Whitney non-parametric test indicated that the BI-GR and BI-PR were comparable in terms of their exposure to Italian, namely AoO-listening, *U* = 127.5, *p* = 0.46, AoO-speaking, *U* = 113.5, *p* = 0.261, LoE, *U* = 260, *p* = 0.598, frequency of Italian use at home, *U* = 168.5, *p* = 0.546 and current Italian language richness, *U* = 140.5, *p* = 0.759.

Regarding the reading assessments, the BI and MONO children within each reading ability group (GR vs. PR) did not significantly differ across the reading measures except for word and text reading accuracy (see Table 1). The PR groups (BI-PR and MONO-PR) attained lower scores than the GR groups (MONO-GR and BI-GR) on all reading measures (see Table 1).

### Experimental tasks

#### Language-dependent measures

##### Nonword repetition

A standardized nonword repetition (NWR) task (Bertelli and Bilancia, 2006) was administered to assess phonological working memory. The task comprised 40 nonwords constructed according to the phonotactic rules in Italian. The nonwords ranged in length from two to five syllables (10 items for each syllable length). This test’s estimated reliability is.67. The children were instructed to listen to the audio-recorded nonwords and to repeat them. Two practice items were provided before testing. Each repeated nonword was scored as correct and received a score of 1 only if it contained all the consonants and vowels of the target in the right order. Otherwise, it was considered incorrect and received a score of zero (maximum score of 40). The standardized scores on the task were used as the outcome measure (NWR _Zscore_).

##### Phonemic awareness

A phoneme Blending test (see Angelelli et al., 2004) was administered to measure the ability to synthesize (blend) speech sounds to make words and nonwords. The test contained a list of 19 words and a list of 19 nonwords, containing five to six phonemes each. Test items were audio-recorded and were presented *via* an animated PowerPoint Presentation. Each item was presented phoneme by phoneme. Children were instructed to listen to the series of phonemes and to repeat aloud the entire word/nonword. Four practice items were presented before the testing of each list. One point was given for each item blended correctly. The outcome measure (PA _Total_) was the percentage of correctly blended words and nonwords.

##### Sentence repetition

To assess the morpho-syntactic production abilities, a shortened version of the Italian LITMUS Sentence Repetition (SA) task was administered (developed and kindly made available by Levorato and Roch, see Marinis and Armon-Lotem, 2015). The task included a total of 30 sentences divided into three levels of syntactic complexity. The sentences targeted various grammatical structures such as past tense and agreement inflections, copula verbs, clitics, clausal agreement, subject, and object relative clauses, Wh-object questions, and short and long passives. The sentences were pre-recorded and presented *via* an animated PowerPoint Presentation. Children listened to the sentences, one at a time, and repeated each sentence verbatim. Two practice items were given to the children before the presentation of the test sentences. In the binary scoring method, the child received a score of 1 if they repeated the whole sentence correctly. Repetitions containing any omission, substitution, or addition of words and/or affixes received a score of 0. In the structural scoring method, each repeated sentence received a score of 1 or 0 based on whether the target grammatical structure was maintained or not. The maximum raw score of each scoring method was 30. The task yielded two outcome measures: the percentage of sentences repeated entirely correctly (SR _Binary_) and the percentage of sentences in which the target grammatical structure was repeated correctly (SR _Structure_).

#### Language independent measures

##### Auditory reaction time

Children completed a computerized Auditory reaction time (RT) task designed by Carioti et al. (2022b). In this task, children were shown a traffic light on a computer screen and were informed that when the traffic light turned green, they would hear some tones (i.e., 440 Hz pure tones). Children were instructed to listen to the tones and to respond as quickly as possible by clicking the mouse. A total of 10 tones were presented randomly. The first two tones were “familiarization trials,” and the subsequent eight tones were the “experimental trials.” For each experimental trial, the RT, in milliseconds, was calculated as the difference between the time of the child’s response (i.e., mouse click) and the time of the tone. The outcome measure (RT) reflected the median RT totaled across the eight trials for each child. This was a control measure to confirm no differences among the groups in auditory RT.

##### Timing anticipation

The Warning-Imperative task (Pagliarini et al., 2020) was administered to investigate the children’s timing anticipation skills. The task consisted of two phases. In the familiarization phase, children listened to a regular sequence of metronome beats with a reference tempo of 80 beats per minute (bpm, inter-onset interval of 750 ms). The beats were 440 Hz pure tones with 8 ms rise and fall times and 200 ms steady-state duration. In the testing phase, children listened to the same regular sequence, which included a pair of distinct beats: a warning beat (WB) and an imperative beat (IB). The WB was created by adding an 880 Hz beep to the basic sound, and its role was to alert the children to tap in time with the incoming IB. Hence, the WB put the children in anticipation mode. Each pair of WB-IB were randomly presented throughout a rhythmic sequence. Each sequence consisted of 10 beats (8 basic beats, a WB, and an IB). Children were presented with the rhythmic sequences 10 times. The first outcome measure was timing error which corresponded to the difference between the median time of the child’s taps on the IB and the actual time of the IB across the 10 trials (Anticipation_Timing error_). The second outcome measure was individual variability which corresponded to the standard deviation of the average timing error of each child (Anticipation_Variability_).

##### Beat synchronization

A computerized tapping task (Carioti et al., 2022b) was employed to evaluate the children’s beat synchronization abilities. There were two conditions: entrainment (paced tapping to regular beats) and free tapping (continued tapping after the beats stop). The rhythmic sequences in the entrainment and free tapping conditions varied in speed. There was a slow rhythmic sequence with a rate of 80 bpm with an inter-onset-interval of 750 ms, and a fast rhythmic sequence with a rate of 100 bpm with an inter-onset-interval of 600 ms. The task consisted of three phases paired with a traffic light signal. A red traffic light marked the familiarization stage in which children were asked to listen to the rhythmic sequence. A yellow traffic light marked the entrainment phase, where children were required to tap along to the beats using the mouse button. Finally, when the red traffic light was on, the beats stopped (no audible tones), and children were required to continue tapping at the rate of the last rhythmic sequence they heard. Rhythmic entrainment trials consisted of 16 beats, so the 16 corresponding taps were recorded. In the free tapping phase, the first 12 taps (implying 12 beats) were recorded.

The task was first administered using the slow rhythmic rate followed by the fast rhythmic rate. Performance on each condition was described using two indices. Timing error indicated how far the children’s taps deviated from the expected tapping rate. Timing error was calculated as the absolute difference between the child’s median inter-tap intervals (ITI) and the target ITI (i.e., 750 ms for the slow rhythmic rate and 600 ms for the fast rhythmic rate). To illustrate, for a slow rhythmic sequence, a child’s median ITI of 700 would result in a timing error of 50 ms (i.e., 700–750 = |-50|). Tapping variability showed how consistent the child’s tapping rate was within a condition. Tapping variability was calculated as the standard deviation of each child’s ITI within a condition. Accordingly, there were eight outcome measures for this task: Entrainment-slow _Timing error_, Entrainment-slow _Variability_, Free Tapping-slow _Timing error_, Free Tapping-slow _Variability_, Entrainment-fast _Timing error_, Entrainment-fast _Variability_, Free Tapping-fast _Timing error_, and Free Tapping-fast _Variability_.

##### Inhibitory control

A cued auditory Go/No-Go paradigm devised by Carioti et al. (2022b) was employed to assess inhibition. The task utilized a pair of stimuli, a 440 Hz low-frequency beat (Go beat) and an 880 Hz high-frequency beat (NoGo beat). The task consisted of two blocks: in the irregular block, the inter-stimulus interval varied between 250 and 1,000 ms, whereas in the regular block, the inter-stimulus interval was constant at 850 ms. The presentation of the stimuli in the regular version resulted in a rhythmic sequence of 60 bpm allowing children to extract a temporal structure to predict the incoming stimulus. According to Carioti et al. (2022b), this design was done to examine the effect of the stimuli’s regularity on task performance. Children were instructed to look at the computer screen and click the mouse as quickly as possible in response to the Go beat and refrain from clicking when they heard the NoGo beat. Children completed six familiarization trials in the first phase of the task (3 Go, 3 NoGo). Next, children completed 24 trials (16 Go, 8 NoGo) of the irregular block, then 24 trials (16 Go, 8 NoGo) of the regular block with a short break in-between. Performance on the Go/NoGo was indexed by d’prime, a discrimination sensitivity index calculated by subtracting the z-transformed false alarm rate from the z-transformed hit rate. This was done separately for irregular (Dprime _Irregular_) and regular (Dprime _Regular_) conditions.

##### Rapid automatized naming

Children completed a computerized rapid automatized naming of shapes task (RAN-shapes; developed by Carioti et al. (2022a). The test consists of three 200 mm × 200 mm matrices that vary in the number of shapes displayed (i.e., cognitive demand) and their perceptual properties such as shape size and background texture (i.e., attentional burden). Five standard shapes were used: heart, star, triangle, square, and circle. Accordingly, Matrix 1 contained 49 shapes printed across a 7 × 7 grid. Matrix 2 contained 100 shapes across a 10 × 10 grid; thus, the shapes looked smaller and closer to each other than Matrix 1. Matrix 3 was the same as Matrix 1 but with an interfering background. In the familiarization phase of the test, children were asked to look at the computer screen and name the individually presented shapes. In the testing phase, the matrices were displayed, one at a time, in a fixed order. Children were instructed to name the shapes in the conventional order (left to right, top to bottom). The number of correctly named shapes in 30 s was calculated for each matrix. The outcome measure (RAN _Total_) was naming speed as indexed by the sum of correctly named shapes across the three matrices.

### Procedure

Each child was tested individually by a research assistant in a quiet room in their school or at the Neurological Institute Carlo Besta. The children participated in a larger research project and were assessed using a battery of tasks administered across three sessions lasting 45 min each. In the first session, non-verbal abilities, forward and backward digit recall, NWR, and word and nonword reading tasks were administered. SR, text reading, comprehension, and PA tasks were administered in the second session. In the third session, an eye-tracking task, RAN, RT, expressive rhythm, inhibitory control, and timing anticipation tasks were administered.

### Statistical analysis

All statistical analyses were performed in R, Version 4.1.2 (R Core Team, 2021). To address the first research question, a series of generalized linear models (GLMs) were run using the *glm* function of the *stats* package (R Core Team, 2021). A total of 19 GLMs were carried out, one for each of the outcome measures of the different tasks. In each model, the outcome measure of interest was included as the dependent variable. Reading ability (GR vs. PR), Bilingualism (MONO vs. BI), and their interaction were entered as the between-subject independent variables. As noted in the Method section, there were no significant differences in chronological age among the groups.

Moreover, the non-verbal IQ was measured using different tools, limiting group comparisons. There were no differences among the BI groups regarding measures of exposure to Italian. Therefore, none of these variables were adjusted for in the models. Before conducting the GLMs, data distribution was inspected, and outliers were removed iteratively with a maximum of two rounds based on boxplots. In cases where the outcome measures (after removing outliers) were not normally distributed, the *fiGRist* function of the *MASS* package (Venables and Ripley, 2002) was used to determine the distribution family that best fits the data.

The following outcome measures: NWR _Zscore_, PA _Total_, RAN _Total_, Anticipation _Timing error_, Anticipation _Variability_, Entrainment-slow _Variability_, Free Tapping-fast _Variability_, Dprime _Irregular,_ and Dprime _Regular_ were modeled using GLMs employing a normal distribution with an identity link function. RT, Entrainment-slow _Timing error_, Free Tapping-slow _Timing error_, Free Tapping-slow _Variability_, Entrainment-fast _Timing error_, Entrainment-fast _Variability_, and Free Tapping-fast _Timing error_ were positively skewed. These variables were modeled with GLMs employing a Gamma distribution with an inverse link function (see Anderson et al., 2012). Finally, the SR _Binary_ and SR _Structure_ scores were negatively skewed and were modeled with a Tobit regression using the *vglm* function of the *VGAM* package (Yee et al., 2015). Analysis of variance using the *Anova* function was performed on the fitted models to determine the significance levels of the main effects and their interactions. Residual diagnostics of the fitted models were checked using the *simulateResiduals* and *plot* functions of the *DHARMa* package (Hartig, 2021).

To address the second research question, Spearman zero-order correlations were calculated between the performance on each of the language-dependent, language-independent measures and reading ability (i.e., word, nonword, and text reading speed and accuracy) and Italian language exposure measures as indexed by LoE. This was done using the *rcor* function of the *Hmisc* package (Harrell, 2021). Statistical thresholds for the two analyses were corrected for multiple comparisons using the false discovery rate (FDR) technique (Benjamini and Hochberg, 1995) at alpha = 0.05.

## Results

### Group comparisons

#### Language dependent measures

##### NWR

Descriptive statistics of the performance of the groups on the language-dependent tasks are shown in Table 2. A GLM was conducted with the NWR _Zscore_ as the dependent variable. Data of 73 children were included after the removal of 1 outlier (1 MONO-GR). A significant main effect of reading ability was found, X^2^(1) = 31.77, *FDR-corrected-p < 0.001*, η^2^ = 0.31, with the PR group (*M* = −2.93, *SD* = 2.26) showing significantly lower NWR scores than the GR group (*M* = −0.37, *SD* = 1.81). The main effects of bilingualism, X^2^(1) = 4.35, *FDR-corrected-p = 0.159*, η^2^ = 0.04, and the interaction term were not significant, X^2^(1) = 0.22, *FDR-corrected-p* = 0.847, η*^2^* = 0.002.

**Table 2:** Groups’ performance on the language-dependent measures.

	Group					
Outcome measures	MONO-GR Mean(SD)	MONO-PR Mean(SD)	BI-GR Mean(SD)	BI-PR Mean(SD)	Uncorrected group differences	FDR-corrected group differences
NWR _Zscore_	0.05(1.92)	−2.38(1.97)	−0.72(1.68)	−3.58(2.46)	PR < GR, BI < MONO	PR < GR
PA _Total_	79.59(10.74)	71.35(16.91)	79.66(13.66)	62.09(17.15)	PR < GR	PR < GR
SR _Binary_	95.37(5.73)	93.33(4.44)	92.50(6.57)	85.46(7.78)	PR < GR, BI < MONO	PR < GR, BI < MONO
SR _Structure_	97.22(3.29)	95.96(2.85)	96.83(4.28)	93.06(4.13)	PR < GR	PR < GR

MONO-GR, monolingual good readers; MONO-PR, monolingual poor readers; BI-GR, bilingual good readers; BI-PR, bilingual poor readers; NWR, nonword repetition; PA, phonological awareness; SR, sentence repetition; FDR, false discovery rate.

##### PA

A GLM with PA _Total_ scores of all 74 children revealed a significant main effect of reading ability, X^2^(1) = 15.97 *FDR-corrected-p < 0.01*, η^2^ = 0.18, whereby the PR groups (*M* = 66.99%, *SD* = 17.41) had a lower phoneme blending accuracy than the GR groups (*M* = 79.63, *SD* = 12.24). The effects of Bilingualism, X^2^(1) = 1.52, *FDR-corrected-p* = 0.608, η^2^ = 0.01, and the interaction between reading ability by bilingualism, X^2^(1) = 0.901, *FDR-corrected-p* = 0.50, η^2^ = 0.02 were not significant.

##### SR

The SR _Binary_ scores of 68 children were analyzed after removing six outliers (1 BI-GR, 5 BI-PR). There was a significant main effect of reading ability on the SR _Binary_ scores, X^2^(1) = 8.59, *FDR-corrected-p < 0.05*, η^2^ = 0.10 such that the PR groups (*M* = 90.44, *SD* = 6.28) repeated sentences less accurately than GR groups (*M* = 93.86, *SD* = 6.93). There was also a main effect of bilingualism, X^2^(1) = 10.01, *FDR-corrected-p < 0.05,* η^2^ = 0.13, with the BI groups (*M* = 90, *SD* = 7.69) showing lower SR _Binary_ scores than the MONO groups (*M* = 94.21, *SD* = 5.14). The interaction between reading ability and bilingualism was not significant, X^2^(1) = 1.96, *FDR-corrected-p* = 0.529, η^2^ = 0.03.

The SR _Structure_ scores of 70 children were analyzed after removing four outliers (4 BI-PR). The SR _Structure_ scores differed across reading ability groups, X^2^(1) = 8.96, *FDR-corrected-p < 0.05*, η^2^ = 0.09. The PR groups (*M* = 94.84, *SD* = 3.64) repeated the target grammatical structures less accurately than the GR groups (*M* = 97.01, *SD* = 3.81). The main effects of bilingualism, X^2^(1) = 1.80, *FDR-corrected-p = 0.476,* η^2^ = 0.04, and the interaction term were not significant, X^2^(1) = 1.32, *FDR-corrected-p* = 0.52, η^2^ = 0.03.

#### Language independent measures

##### Auditory reaction time

Group data for the language-independent measures are shown in Table 3. RTs of 70 children were analyzed after removing 4 outliers (3 BI-PR, 1 MONO-PR). The RTs did not differ significantly across the reading ability groups, X*^2^*(1) = 1.84, *FDR-corrected-p* = 0.476, η^2^ = 0.029, nor the language groups X^2^(1) = 1.09, *FDR-corrected-p* = 0.540, η^2^ = 0.017, The reading ability by bilingualism interaction was not significant, X^2^(1) = 0.03, *FDR-corrected-p* = 0.936, η^2^ = 0.950.

**Table 3:** Groups’ performance on the language-independent measure.

	Group					
Outcome measures	MONO-GR Mean(SD)	MONO-PR Mean(SD)	BI-GR Mean(SD)	BI-PR Mean(SD)	Uncorrected group differences	FDR-corrected group differences
RT (ms)	261.43(87.44)	292.73(87.44)	246.25(59.85)	267(83.20)	NO	NO
Anticipation _Timing error_	57.68(64.25)	86.05(104.13)	23.94(113.21)	123.83(140.61)	PR > GR	NO
Anticipation _Variability_	97.47(35.19)	102.23(58.65)	96.08(52.81)	109.79(53.82)	NO	NO
Entrainment-slow _Timing error_	30.85(22.74)	37.81(18.03)	30.34(17.01)	48.94(30.64)	PR > GR	NO
Entrainment-slow _Variability_	55.05(32.81)	62.55(29.41)	57.26(27.90)	62.42(32.94)	NO	NO
Free Tapping-slow _Timing error_	71.35(64.80)	114.05(65.94)	89.69(65.25)	136.31(82.74)	PR > GR	NO
Free Tapping-slow _Variability_	44.87(29.39)	38.37(19.75)	42.78(23.06)	48.07(29.39)	NO	NO
Entrainment-fast _Timing error_	15.52(12.97)	16(8.74)	14.56(9.12)	24.04(16.67)	NO	NO
Entrainment-fast _Variability_	38.06(11.65)	49.69(11.65)	38.64(17.57)	51.27(22.27)	PR > GR	PR > GR
Free Tapping-fast _Timing error_	38.15(32.75)	49.40(23.76)	38.15(24.56)	52.60(32.88)	NO	NO
Free Tapping-fast _Variability_	36.53(17.17)	37.48(14.45)	26.79(14.43)	36.08(14.20)	NO	NO
Dprime _Irregular_	1.87(0.99)	1.57(0.56)	1.89(0.95)	1.22(0.57)	GR > PR	NO
Dprime _Regular_	1.44(0.91)	1.33(0.70)	1.97(0.73)	1.22(1.02)	GR > PR	NO
RAN _Total_	105.50(18.99)	89.94(18.15)	99.70(20.15)	94.21(17.18)	PR < GR	NO

MONO-GR, monolingual good readers; MONO-PR, monolingual poor readers; BI-GR, bilingual good readers; BI-PR, bilingual poor readers; RT, reaction time; RAN, rapid automatized naming; FDR, false discovery rate.

##### Timing anticipation

**Figure 1:**
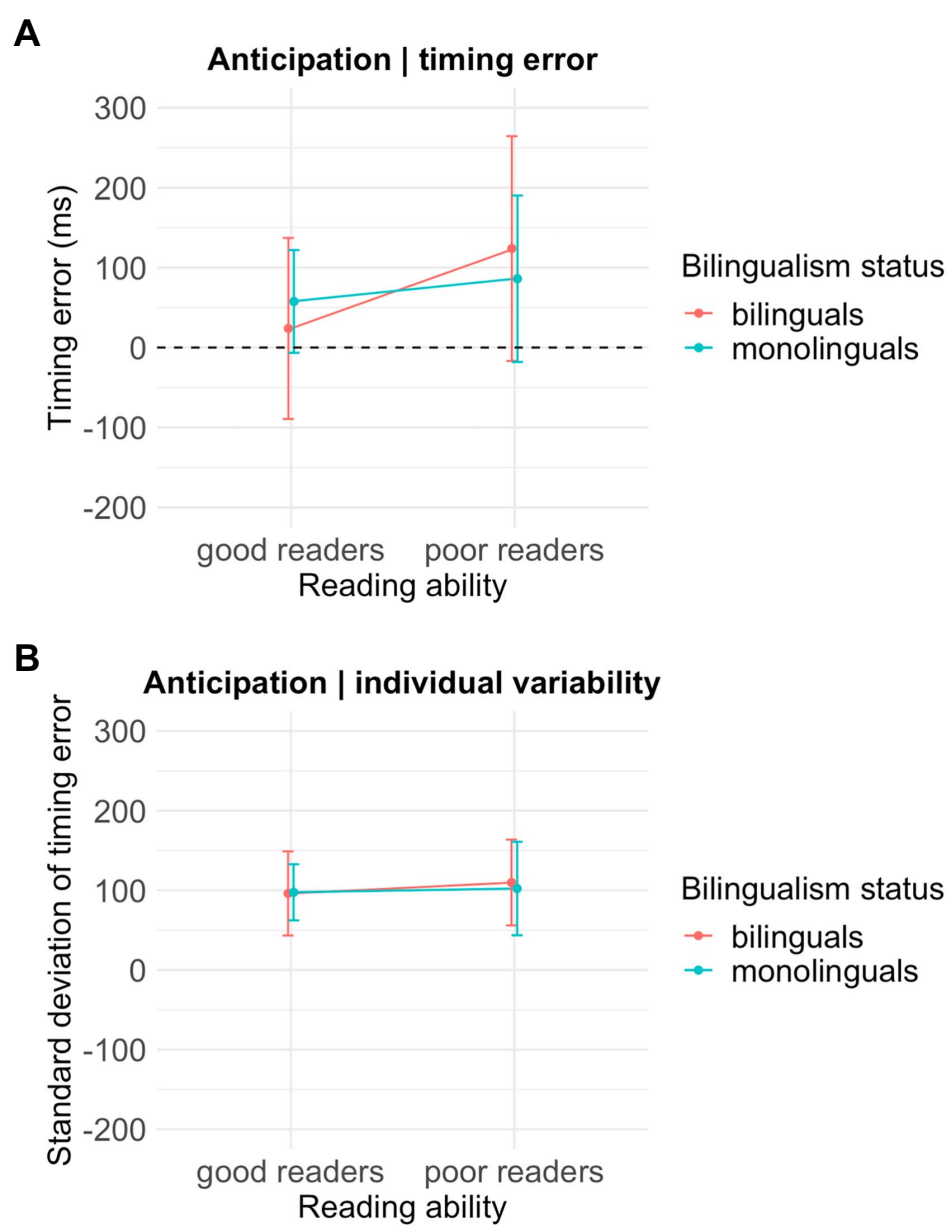
Group performance on the timing anticipation task. **(A)** The horizontal line represents the time of the imperative beat (IB). Timing error reflects the difference between the group’s average tapping time and the time of the IB. **(B)** Average variability (standard deviations) of the timing error is shown.

Anticipation _Timing error_ measures of 56 children were analyzed after removing eight outliers (2 MONO-GR, 4 BI-GR, 2 BI-PR, and 10 missing values). The Anticipation _Timing error_ appeared to differ across the reading ability groups, X^2^(1) = 4.54, *uncorrected-p* < 0.05, η^2^ = 0.08 with the PR groups (*M* = 101.44 ms, *SD* = 119.23) showing a greater timing error in anticipating the IB than the GR groups (*M* = 40.22 ms, *SD* = 92.84). Figure 1A illustrates that the PR groups tended to tap farther away from the IB than the GR groups. This effect however did not survive FDR corrections (*FDR-corrected-p* = 0.159). The Anticipation _Timing error_ did not differ across the bilingualism status, X^2^(1) = 0, *FDR-corrected-p* = 0.996, η^2^ = 0 and the interaction term was not significant, X*^2^*(1) = 1.54, *FDR-corrected-p* = 0.215, η^2^ = 0.03.

The Anticipation _Variability_ scores (i.e., individual variability in the Anticipation _Timing error_) of 64 were analyzed after removing two outliers (1 MONO-GR, 1 MONO-PR, eight missing values). The Anticipation _Variability_ did not differ significantly across reading ability, X^2^(1) = 0.53, *FDR-corrected-p* = 0.648, η^2^ = 0.008 nor Bilingualism status, X^2^(1) = 0.05, *FDR-corrected-p* = 0.936, η^2^ = 0, and the interaction term was not significant, X^2^(1) = 0.12, *FDR-corrected-p* = 0.914, η^2^ = 0.002. See Figure 1B.

##### Beat synchronization

**Figure 2:**
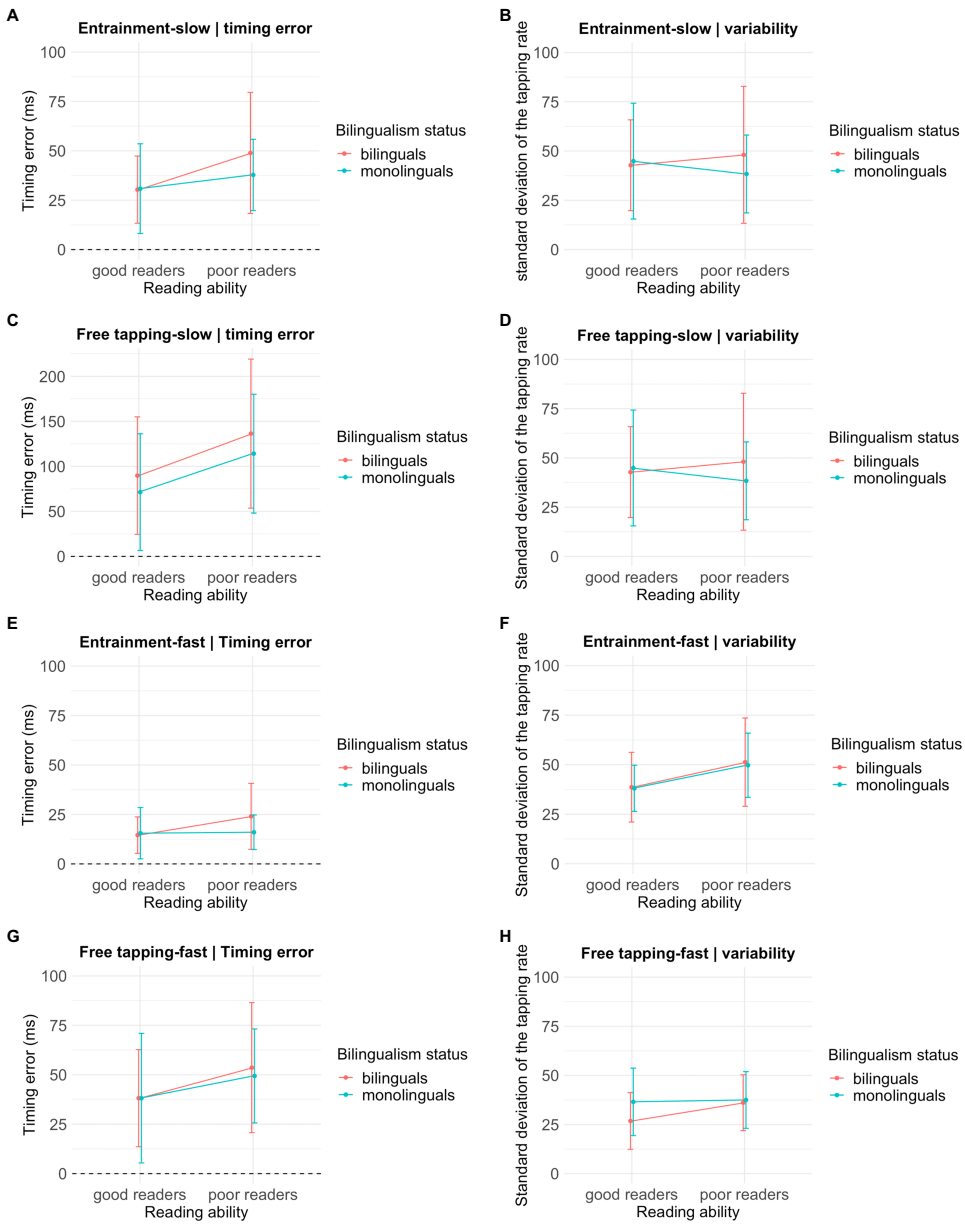
Group performance on the beat synchronization task. **(A,C,E,G)** The horizontal line represents the expected tapping rate. Timing error reflects the difference between the group’s tapping rate and the expected tapping rate for each condition. **(B,D,F,H)** Average variability (standard deviations) of the tapping rate is shown.

For the Entrainment-slow _Timing error_, observations of 63 children were included following the removal of 4 outliers (1 MONO-GR, 1 MONO-PR, 2 BI-PR, and seven missing values). The Entrainment-slow _Timing error_ differed across reading ability conditions, X^2^(1) = 5.34, uncorrected-*p* < 0.05, η^2^ = 0.08. As displayed in Figure 2A, the tapping rate of the PR groups (*M* = 42.97, *SD* = 24.8) deviated from the expected tapping rate (represented by the horizontal line at zero) to a greater degree than the GR groups (*M* = 30.56, *SD* = 19.36). That is, the PR groups made larger timing errors. This group difference disappeared after FDR corrections (*FDR-corrected-p* = 0.114). The Entrainment-slow _Timing error_ did not differ across bilingualism status, X^2^(1) = 0.71, *FDR-corrected-p* = 0.608, η^2^ = 0.01, and the bilingualism by reading ability interaction was not significant, X^2^(1) = 0.58, *FDR-corrected-p* = 0.647, η^2^ = 0.91.

Regarding inter-subject tapping variability, Entrainment-slow _Variability_ measures of 68 children were included following the removal of two outliers (1 MONO-GR, 1 BI-PR, and four missing values). A GLM with the Entrainment-slow _Variability_ revealed no significant effect of reading ability X^2^(1) =0.69, *FDR-corrected-p* = 0.608, η^2^ = 0.01, bilingualism, X^2^(1) = 0.02, *FDR-corrected-p* = 0.936, η^2^ = 0, or their interaction X^2^(1) = 0.02, *FDR-corrected-p* = 0.608, η^2^ = 0 on tapping consistency (see Figure 2B).

The Free Tapping-slow _Timing error_ measures of 68 children were analyzed (6 missing values). A GLM with the Free Tapping-slow _Timing error_ revealed a significant difference across reading ability groups, X^2^(1) = 6.28, un-corrected *p* < 0.05, η^2^ = 0.10. As shown in Figure 2C, the PR groups (*M* = 124.49, *SD* = 73.92) demonstrated greater deviance from the expected tapping rate relative to the GR groups (*M* = 81.54, *SD* = 64.78). However, this group difference disappeared once FDR corrections were applied (*FDR-corrected-p* = 0.094). The Free Tapping-slow _Timing error_ did not differ significantly with respect to bilingualism status, X^2^(1) = 1.24, *FDR-corrected-p* = 0.529, η^2^ = 0.02, and the interaction term was not significant X^2^(1) = 0.151, *FDR-corrected-p* = 0.896, η^2^ = 0.89.

Free-Tapping-slow _Variability_ scores of 65 children were analyzed after the removal of three outliers (2 MONO-GR, 1 BI-PR, six missing values). There was no significant effect of reading ability X^2^(1) = 0.004, *FDR-corrected-p* = 0.969, η^2^ = 0, bilingualism X^2^(1) = 0.305, *FDR-corrected-p* = 0.784, η^2^ = 0.01 or their interaction X^2^(1) = 0.608, *FDR-corrected-p* = 0.608, η^2^ = 0.99 on tapping consistency (see Figure 2D).

The Entrainment-fast _Timing error_ scores of 56 children were included after removing six outliers (1 MONO-GR, 4 MONO-PR, 1 BI-GR, 3 BI-PR, and nine missing values). The Entrainment-fast _Timing error_ did not differ across reading ability, X^2^(1) = 2.59, *FDR-corrected-p* = 0.385, η^2^ = 0.05 or bilingualism status, X^2^(1) = 0.74, *FDR-corrected-p* = 0.608, η^2^ = 0.01. The interaction term was also not significant, X^2^(1) = 1.29, *FDR-corrected-p* = 0.259, η^2^ = 0.94 (see Figure 2E).

The Entrainment-fast _Variability_ scores of 64 children were analyzed after removing four outliers (1 MONO-GR, 3 BI-PR, six missing values). The Entrainment-fast _Variability_ differed across reading groups, X^2^(1) = 7.82, FDR-corrected *p* < 0.05, η^2^ = 0.12 but not across bilingualism conditions, X^2^(1) = 0.06, *FDR-corrected-p* = 0.936, η^2^ = 0. The bilingualism by reading ability interaction was not significant, X^2^(1) = 0.013, *FDR-corrected-p* = 0.943, η^2^ = 0 (Figure 2F).

The Free Tapping-fast _Timing error_ scores of 62 children were included following removing four outliers (2 MONO-PR, 1 BI-GR, 1 BI-PR, and eight missing values). There were no significant main effects of reading ability, X^2^(1) = 3.15, *FDR-corrected-p* = 0.292, η^2^ = 0.06, bilingualism, X^2^(1) = 0.06, *FDR-corrected-p* = 0.936, η^2^ = 0, or their interaction, X^2^(1) = 0.04, *FDR-corrected-p* = 0.936, η^2^ = 0.94 on the Free Tapping-fast _Timing error_ (see Figure 2G).

Free-Tapping-fast _Variability_ scores of 66 children were analyzed following the removal of outliers (1 MONO-PR, 1 BI-GR, six missing values). Similarly, there were no significant main effects of reading ability, X^2^(1) = 1.85, *FDR-corrected-p* = 0.476, η^2^ = 0.03, bilingualism, X^2^(1) = 2.32 or *FDR-corrected-p* = 0.408, η^2^ = 0.04 and their interaction, X^2^(1) = 1.76, *FDR-corrected-p* = 476, η^2^ = 0.93 on the Free-Tapping-fast _Variability_ scores (see Figure 2H).

##### Inhibitory control

The Dprime _Irregular_ scores of 66 children were analyzed after removing 1 outlier (1 BI-GR, seven missing values). A GLM with Dprime _Irregular_ scores revealed a significant main effect of reading ability, *X^2^*(1) = 4.29, *uncorrected-p* < 0.05, η^2^ = 0.06, such that the Dprime _Irregular_ scores of the PR groups (*M* = 1.28, *SD* = 0.85) were lower than the GR Groups (*M* = 1.72, *SD* = 0.85). This group difference disappeared after FDR corrections (*FDR-corrected-p* = 0.159). The main effects of bilingualism, X^2^(1) = 1.07, *FDR-corrected-p* = 0.540, η^2^ = 0.02, and the interaction between reading ability and bilingualism, X^2^(1) = 2.38, *FDR-corrected-p* = 0.408, η^2^ = 0.03 were not significant.

The Dprime _Regular_ scores were available for 66 children. The GLM revealed significant main effects of reading ability, X^2^(1) = 5.77, *uncorrected-p* < 0.05, η^2^ = 0.08, with the PR groups (*M* = 1.42, *SD* = 0.58) showing lower Dprime _Regular_ scores than the GR groups (*M* = 1.88, *SD* = 0.96). This group difference disappeared after FDR corrections (*FDR-corrected-p* = 0.110). The main effects of bilingualism, X^2^(1) = 0.557, *FDR-corrected-p* = 0.647, η^2^ = 0 and the interaction term were not significant X^2^(1) = 0.794, *FDR-corrected-p* = 0.608, η^2^ = 0.01.

##### RAN

The RAN _Total_ scores were available for 68 children. The RAN _Total_ scores differed across reading groups X^2^(1) = 5.32, un-corrected *p < 0.05,* η^2^ = 0.08. The PR groups (*M* = 91.82, *SD* = 19.59) had lower naming speed than the GR groups (*M* = 102.28, *SD* = 17.58). This effect did not survive FDR corrections(*FDR-corrected-p* = 0.114). The main effects of bilingualism, X^2^(1) = 0.05, *FDR-corrected-p* = 0.936, η^2^ = 0 and the interaction between reading ability and bilingualism, 4×^2^(1) = 1.20, *FDR-corrected-p* = 0.529, η^2^ = 0.02 were not significant.

### Relationship between language processing, cognitive abilities, and reading

The results of the zero-order and FDR-corrected correlational analyses are presented in Supplementary Table S1. First, the relationship between **language-dependent tasks** and reading was examined. There was a weak positive correlation between NWR and word reading speed (*r* = 0.36, *FDR-corrected p* < 0.001). There were also moderate positive association between NWR and word reading accuracy (*r* = 0.50, *FDR-corrected p* < 0.001), nonword reading accuracy (*r* = 0.45, *FDR-corrected p* < 0.001), text reading speed (*r* = 0.45, *FDR-corrected p* < 0.001) and text reading accuracy (*r* = 0.50, *FDR-corrected p* < 0.001).

There were moderate and positive correlations between PA and word (*r* = 0.4, *FDR-corrected p* < 0.001) and nonword reading accuracy (*r* = 0.39, *FDR-corrected p* < 0.05). PA correlated with word, nonword, reading speed, and text reading accuracy. These latter correlations were weak (*r* ≤ 0.3) and did not survive corrections for multiple comparisons (all *FDR-corrected p* > 0.05). The correlations between SR and word, nonword, and text reading speed were weak and did not survive corrections for multiple comparisons (all *FDR-corrected p-values* > 0.05).

Second, we examined the relationship between performance on **language-independent tasks** and reading. No correlations were found between performance on the timing anticipation task and any of the reading measures. All of the correlations between beat synchronization variables and reading measures were weak (*r* ≤ 0.3), and did not survive corrections for multiple comparisons (all *FDR-corrected p-values* > 0.05). We observed a correlation between inhibition control and reading speed (*r* = 0.28, *FDR-corrected p* < 0.05), as well as text reading accuracy (*r* = 0.34, *FDR-corrected p* < 0.01). There were weak to moderate correlations between performance on RAN and the speed of reading words (*r* = 0.37, *FDR-corrected p* < 0.05) and nonwords (*r* = 0.44, *FDR-corrected p* < 0.05). Lastly, timing error in free tapping to slow rhythms negatively correlated with NWR accuracy (*r* = −0.35, *FDR-p* < 0.05).

Finally, we investigated the relationship between performance on the tasks and Italian language exposure (as indexed by LoE in months). Only one correlation emerged, with children with longer exposure to Italian also having higher RAN scores (*r* = 0.26, FDR*-corrected p* < 0.05).

## Discussion

The current study investigated the potential of language-dependent and language-independent approaches for identifying dyslexia in children with diverse linguistic backgrounds. We found that poor readers underperformed good readers on language-dependent tasks, including NWR, PA, and SR. In contrast, there were no significant group differences between poor and good readers in their performance in the language-independent tasks, including timing anticipation, beat synchronization (except for tapping variability at fast rhythms), inhibition control, and rapid automatized naming. Monolinguals and bilinguals only differed in their performance on the SR task. Furthermore, there were multiple weak to moderate correlations between some language-dependent processing measures (nonword repetition and phonological awareness), language-independent cognitive measures (inhibition control and naming speed), and reading. Performance on the language-dependent and language-independent tasks (apart from rapid automatized naming) did not correlate with the length of exposure to Italian. We discuss these findings in more detail below.

### Performance on the language-dependent and language-independent tasks

To address the first research question, we examined whether performance on the language-dependent and language-independent tasks differed by reading ability and bilingual experience and whether these two factors interact. Concerning the language-dependent tasks, as predicted, we found a significant effect of reading ability on NWR performance. Poor readers, both monolinguals and bilinguals, scored below good readers on the NWR task, confirming that phonological processing is an area of difficulty for children with reading difficulties (Ehrhorn et al., 2020; Vender et al., 2020; Vender and Melloni, 2021). In line with our predictions, bilingualism status did not affect NWR performance. This result corroborates previous studies showing that bilingual Italian-speaking children with diverse linguistic backgrounds perform similarly to their monolingual peers in NWR tasks (Guasti et al., 2013; Vender et al., 2016, 2020; Vender and Melloni, 2021). Notably, the NWR test used in this study was constructed following the Italian phonotactic system known for its simple phonemic and syllabic structures (Vender et al., 2020).

Consistent with our predictions, we found a significant effect of reading ability but no effect of bilingualism on PA. Monolingual and bilingual poor readers exhibited lower phoneme blending accuracy than good readers. These findings align with studies revealing PA deficits in monolingual (Menghini et al., 2010; Tobia and Marzocchi, 2014) and bilingual Italian-speaking children with dyslexia (Vender and Melloni, 2021). The comparable performance of monolinguals and bilinguals in PA has also been documented (Guron and Lundberg, 2003; Vender and Melloni, 2021). The Italian language has a simple phonotactics system and shallow orthography characterized by highly transparent grapheme-phoneme correspondences (Seymour, 2005). Acquiring PA skills is therefore expected to take place easily and rapidly after sufficient exposure to Italian (Vender et al., 2020). An alternative explanation is related to the nature of PA as a metalinguistic ability that is “shared across languages, and therefore is expected to transfer between languages” (p.444, Wawire and Kim, 2018). This view is supported by studies showing that PA skills in L1 and L2 are correlated (e.g., Cisero and Royer, 1995; Bialystok et al., 2009; Kim, 2009; Wawire and Kim, 2018; Krenca et al., 2020). This cross-language transfer is evident even when PA skills are still developing (Kwakkel et al., 2021).

As predicted, reading difficulties greatly affected SR performance. Within the monolingual and bilingual groups, poor readers scored below the good readers on the SR task. We found these group differences across the binary and structural scoring methods. Previous studies have documented SR deficits in monolingual children with dyslexia (Plaza et al., 2002; Moll et al., 2015; Dosi and Koutsipetsidou, 2019). Given that SR requires interaction between verbal memory resources and linguistic knowledge (Riches, 2012; Marinis and Armon-Lotem, 2015), the poor readers’ difficulty with SR could reflect language difficulties or limitations in verbal short-term memory capacity. When binary scores (percentage of correct identical repetitions) were used, the bilingual children, with and without reading difficulties, underperformed their monolingual peers. This bilingual disadvantage in SR has been reported previously (e.g., Chiat et al., 2013; Komeili and Marshall, 2013; Meir et al., 2016; Fleckstein et al., 2018), suggesting that L2 knowledge is involved in SR. There is evidence that L2 expressive vocabulary and language exposure predict SR in school-age bilingual TD children (Pratt et al., 2021). Accordingly, the lower SR performance of bilingual children may be linked to their generally lower Italian language proficiency. We did not observe differences between monolingual and bilingual children when we used the structural scoring method. This is unsurprising given that less penalizing criteria employed by this scoring method.

Overall, the group comparisons on the language-dependent tasks suggest that NWR, PA, and SR tasks are sensitive to reading difficulties. While SR performance is influenced by the level of L2 proficiency, NWR and PA tasks are not. These findings suggest that language-dependent tasks that tap into phonological processing abilities such as PA and NWR may be suitable for disentangling good readers from poor readers, irrespective of their bilingual experiences. On the other hand, tasks that tap into general linguistic processing, such as SR, may require a certain level of L2 language knowledge. Therefore, the bilingual child’s L2 proficiency and exposure should be taken into consideration when interpreting their SR scores. Our study’s results suggest that using a lenient SR scoring method that focuses on grammatical rather than general language knowledge may be more suitable and less sensitive to the child’s L2 proficiency.

In contrast to our predictions, there were no effects of reading ability on the performance on any language-independent tasks except for one beat synchronization measure, i.e., entrainment to fast rhythms. Compared to good readers, poor readers displayed significantly greater variability (i.e., less consistency) in maintaining the target tapping rate during entrainment to fast rhythms. Such beat synchronization difficulties have been previously reported in monolingual children with dyslexia or DLD (Wolff, 2002; Thomson and Goswami, 2008; Corriveau and Goswami, 2009). Our study extends this evidence by showing that both monolingual and bilingual poor readers show timing imprecision when synchronizing motor behavior to external auditory rhythms. Although none of the remaining language-independent measures yielded significant group differences, we identified some trends in the data. In the timing anticipation task, monolingual and bilingual poor readers tended to make larger timing errors in anticipating the imperative beat relative to good readers. On average, poor readers anticipated the beat after 100 milliseconds of its occurrence, whereas good readers anticipated the beat within 40 milliseconds. Moreover, bilingual and monolingual poor readers had lower dprime scores than good readers in the regular and the irregular versions of the auditory Go/No-Go task. Lastly, we observed that the average scores of monolingual and bilingual poor readers were generally lower than good readers in RAN. Given that children with dyslexia have been shown to have a delay in timing anticipation (Pagliarini et al., 2020), inhibition control (Reiter et al., 2005; Doyle et al., 2018), and RAN (Carioti et al., 2022a), it will be worth examining these observed trends further. There were no differences between monolingual and bilingual children in the language-independent tasks. This finding aligns with our prediction and indicates that performance on these tasks is not influenced by L2 proficiency/knowledge.

### Relationship between language-dependent tasks, language-independent tasks, and reading

To address the second research question, we examined how language-dependent processing, language-independent cognitive abilities, and reading skills correlate. As predicted, we found weak to moderate correlations between NWR, PA, and reading. Higher NWR and PA scores were associated with better reading performance. This relationship highlights the role of phonological processing in reading acquisition in monolingual and bilingual children (Snowling et al., 2003; Melby-Lervåg et al., 2012a; Carroll et al., 2014; Vender et al., 2020).

In contrast to our predictions, there was no association between timing anticipation and any of the reading measures. This result contrasts the findings of Pagliarini et al. (2020), who reported a moderate negative association between anticipation measures (timing error and individual variability) with reading speed and accuracy. We speculate that the discrepancy in results may be due to the task’s difficulty. The anticipation task requires dual inhibition. First, children should refrain from tapping to each beat; second, when they hear the two different beats, they must refrain from tapping to the first beat (the warning beat) and prepare to tap to the second one (the imperative beat). For some children, these requirements may be challenging, which is reflected in the high variability of the anticipation measures. Moreover, Pagliarini et al. included only monolingual children with a diagnosis of dyslexia and with severe reading problems. The diagnostic criteria followed by Pagliarini et al. were also more strict than the criteria adopted in our study. Further research is needed to investigate the link between anticipation skills and reading.

In contrast to our predictions, none of the beat synchronization measures correlated with reading. However, we uncovered a correlation between beat synchronization and NWR. Greater timing errors in free tapping at slow rhythms were associated with lower NWR accuracy. It is suggested that rhythm processing requires the perception of the temporal structure of sounds. Hence, imprecise perception of auditory cues to rhythm in speech diminish the quality of phonological encoding and representations, leading to delays in phonological awareness and adversely affecting reading development (Goswami et al., 2002, 2010; Huss et al., 2011; Ozernov-Palchik and Patel, 2018).

In line with our hypothesis, there were several weak positive correlations between inhibition control and reading. The observed pattern was that better inhibition control was associated with faster text reading speed and higher text reading accuracy. Fluent and accurate reading requires focusing on relevant visual information, ignoring and filtering out irrelevant information, and maintaining speech sounds active and protected from interference in working memory while other reading stages are completed (Doyle et al., 2018). Moreover, higher phoneme blending skills were associated with better inhibition control in the regular Go/No-Go task. As indicated by Carioti et al. (2022b), the stimulus arrival is predictable due to the regularity in which both Go and No-Go beats are delivered. We, therefore, speculate that the correlation between regular Go/No-Go task and PA is mediated by rhythm.

As predicted, there were weak to moderate positive correlations between RAN and reading fluency measures, supporting several previous findings (see Araújo and Faísca, 2019 for a review). RAN is multi-componential and involves the coordination of several sub-processes, such as attentional, phonological, orthographic, memory, motoric, and articulatory processes. Any of these processes could drive its relationship with reading (Wolf et al., 2000).

Finally, performance on the language-dependent and language-independent tasks did not correlate with the length of exposure to Italian. This finding reinforces the idea that these tasks do not require proficient levels in L2. However, performance on RAN has a weak correlation with the length of exposure to Italian, as children who had more prolonged exposure to Italian also had higher RAN scores. However, Italian lexical knowledge is necessary to complete the task.

### Clinical implications, limitations, and future directions

This study was motivated by the lack of appropriate assessment tools and the risk of dyslexia misdiagnosis among bilingual children speaking Italian as their L2. The observed patterns in this study suggest that some of the tasks used here pose a difficulty for poor readers irrespective of their level of proficiency in their L2, i.e., Italian. In particular, poorer performance on the language-dependent tasks, including NWR and PA, and on some language-independent tasks (entrainment at fast rhythms) may likely indicate a reading difficulty. Although performance on the SR tasks was sensitive to reading difficulty, it also depends on the child’s Italian language proficiency. Therefore, the poor performance of bilingual children on the SR tasks should be interpreted in light of their L2 exposure pattern. Although these findings are promising, they require further validation and replication. This is due to a set of limitations which are discussed below.

Using monolingual norms in evaluating bilingual children may lead to an over-diagnosis of language learning disorders such as dyslexia (Connor and Boskin, 2001; Bedore and Peña, 2008). In this study, the classification of children as good or poor readers was determined using standardized Italian reading tests, which were normed for monolingual children. This also reflects the standard clinical practice in Italy, where equivalent criteria are used for diagnosing dyslexia in monolingual and bilingual children. One recommendation is to adapt monolingual norms when assessing bilingual children using more restrictive cut-off points. These cut-offs should be determined according to the language dominance of bilingual children.

It was impossible to control all the potential confounding variables in the present study. For instance, we did not find significant differences in non-verbal IQ or socioeconomic status (as indexed by maternal education) among all groups or in measures of Italian language exposure among bilingual groups. So, their inclusion in the models was not justified. We cannot exclude the possibility that these variables may have an impact, and it is recommended to consider them in future studies.

Many of the models conducted within this study yielded small effect sizes and nonsignificant results. For our sample size (*N* = 74), and a fixed alpha level at.05, post-hoc power calculations revealed that small (*f*^2^ = 0.02), medium (*f*^2^ = 0.15), and large (*f*^2^ = 0.35) group differences were detectable with a with power estimates of 0.17, 0.84, and 0.99, respectively. This suggests the study had adequate power to detect medium and large group differences but inadequate power to detect smaller group differences. Moreover, post-hoc power analyses revealed that small (*r* = 0.1), medium (*r* = 0.3), and large (*r* = 0.5) correlation coefficients were detectable with power estimates of.14, 0.67, and.99, respectively. Hence, our study had adequate power to detect strong correlations but inadequate power to detect weak and moderate correlations. Replication of the results with larger sample size is therefore necessary. Importantly, the number of trials in many of the tasks was low, especially in the rhythmic tasks. This may have increased the likelihood of spurious results. The design of the tasks could be improved by adding more trials to each tested condition.

The findings suggest that language-dependent processing tasks and beat synchronization at fast rhythms are impaired in poor readers with linguistically diverse backgrounds. This finding is based on group differences in the average scores on these tasks. Future studies should determine the usefulness of these tasks in clinical settings by analyzing diagnostic accuracy. This will provide information about the reliability of the tasks in correctly identifying children with reading difficulties (i.e., sensitivity) and excluding those without reading difficulties (i.e., specificity). It is also necessary to establish the psychometric measures for these tasks.

Another aspect to consider is the well-known multi-componential nature of developmental dyslexia as a learning disorder (see Pennington, 2006; McGrath et al., 2020 for reviews). Dyslexia is characterized by several deficits from both a behavioral (e.g., Ramus, 2003) and a neurofunctional point of view (e.g., Danelli et al., 2017). This indicates the need to investigate whether our language-independent tasks can distinguish between good and poor readers using a multi-level approach. This is something that we cannot apply in the present study but may be a promising further line of research.

Future studies may investigate the relationship between language processing, cognitive abilities, and reading by employing hierarchical regression analysis. This is to examine these abilities’ independent and shared contributions to reading development. It will be beneficial to explore how language-dependent processing and language-independent cognitive abilities measured during pre-school age predict later reading development/impairment in the monolingual and bilingual populations. Such data will enhance our understanding of the underlying mechanisms of reading development.

## Conclusion

To summarize, language-dependent tasks discriminated between good and poor readers. In contrast, language-independent tasks (except for entrained tapping at a fast rhythm) did not. Furthermore, the monolingual and bilingual children performed similarly on all tasks except for SR. Performance on RAN was associated with LoE to Italian. Some language-dependent tasks (NWR, PA) and language-independent tasks (inhibition control, RAN) correlated with reading, suggesting that performance on these tasks and reading share underlying cognitive mechanisms. The results highlight the potential of the PA, NWR, SR, and entrainment to fast rhythms for identifying the risk of dyslexia in bilingual populations. Future research is needed to replicate these findings and establish these tasks’ diagnostic accuracy.

## Data availability statement

The datasets presented in this study can be found in online repositories. The names of the repository/repositories and accession number(s) can be found below: the datasets analyzed for this study will be made available on Open Science Framework website (https://osf.io/q5t4k/).

## Ethics statement

The studies involving human participants were reviewed and approved by Research Ethics Committee at the University of Milano-Bicocca (n.461; 7/06/2019) and the Foundation IRCCS Neurological Institute Carlo Besta (n.02; 2/4/2022). Written informed consent to participate in this study was provided by the participants’ legal guardian/next of kin.

## Author contributions

MG, NS, MC, and DC contributed to the study’s conception and design. MC collected the data. DS, EG, and MS helped recruit participants and perform the cognitive assessment. JT and DC conducted the statistical analyses. JT wrote the manuscript, revised the manuscript, and responded to the reviewers’ comments. JT, MG, and DC contributed to refining and proofreading manuscript drafts. All authors contributed to the article and approved the submitted version.

## Funding

This project received funding from the European Union’s Horizon 2020 program for research and innovation under the Marie Skłodowska Curie grant agreement no. 765556 (Principal Investigator: MG).

## Conflict of interest

The authors declare that the research was conducted in the absence of any commercial or financial relationships that could be construed as a potential conflict of interest.

## Publisher’s note

All claims expressed in this article are solely those of the authors and do not necessarily represent those of their affiliated organizations, or those of the publisher, the editors and the reviewers. Any product that may be evaluated in this article, or claim that may be made by its manufacturer, is not guaranteed or endorsed by the publisher.

## Footnotes

^1^While all poor readers recruited in this study have below-average reading scores, not all of them have an official diagnosis of dyslexia.
