# Supplementary material for: Identifying the risk of dyslexia in bilingual children: The potential of language-dependent and language-independent tasks
Source: Front Psychol. 2022 Nov 24;13:935935. doi: 10.3389/fpsyg.2022.935935 (PMC9730291; doi:10.3389/fpsyg.2022.935935)
Supplement: Supplementary file 1 [file Table_1.pdf]

Supplementary Table 1. *Correlations between language-dependent, language-independent, and reading tasks*

		1	2	3	4	5	6	7	8	9	10	11	12	13	14	15	16	17	18	19	20	21	22	23	24	25
1	NWR $_{\text{zscore}}$	-	.36[a]	.54[c]	.47[c]	.2	-.14	.05	-.28	.08	-.35[a]	-.08	-.15	-.16	-.16	-.02	.11	.26	.12	.36[a]	.5[c]	.32	.45[c]	.44[c]	.5[c]	.01
2	PA $_{\text{total}}$	.36[b]	-	.07	.23	-.04	-.14	.23	-.33	.03	-.26	-.08	-.21	-.11	-.07	-.05	.28	.36[a]	.31	.27	.4[a]	.27	.39[a]	.31	.25	.11
3	SR $_{\text{Binary}}$	.54[c]	.07	-	.81[c]	-.02	-.13	-.04	-.12	.06	-.22	-.04	-.23	-.26	-.12	.05	.06	.13	.08	.25	.23	.21	.02	.22	.21	.08
4	SR $_{\text{Structure}}$	.47[c]	.23	.81[c]	-	-.01	-.14	0	-.12	.06	-.13	-.01	-.26	-.25	-.18	-.06	.2	.18	.14	.3	.28	.25	.06	.31	.18	.1
5	RT	.2	-.04	-.02	-.01	-	.24	.12	.08	.24	.09	-.01	.03	.24	-.08	.33	-.16	-.14	-.2	-.12	-.07	-.3	-.05	-.09	-.07	-.03
6	Anticipation $_{\text{Timing error}}$	-.14	-.14	-.13	-.14	.24	-	.22	.16	.05	.01	.06	.2	.01	.18	.01	-.26	-.22	-.14	-.15	-.15	-.21	-.23	-.13	-.16	-.18
7	Anticipation $_{\text{Variability}}$	.05	.23	-.04	0	.12	.22	-	-.08	.11	-.18	.11	.12	.04	-.14	.03	.03	.01	-.01	-.02	-.05	-.15	-.05	-.11	-.12	-.11
8	Entrainment-slow $_{\text{Timing error}}$	-.28[a]	-.33[b]	-.12	-.12	.08	.16	-.08	-	-.19	.13	.14	-.04	.03	.22	0	-.34	-.4	-.17	-.25	-.34	-.2	-.21	-.32	-.35	-.12
9	Entrainment-slow $_{\text{Variability}}$	.08	.03	.06	.06	.24	.05	.11	-.19	-	-.06	.13	.13	.01	-.13	.16	.06	.11	.23	.02	.04	-.04	-.05	0	-.06	.01
10	Free Tapping-slow $_{\text{Timing error}}$	-.35[b]	-.26[a]	-.22	-.13	.09	.01	-.18	.13	-.06	-	.1	.19	.07	.07	.08	-.15	-.28	-.14	-.28	-.16	-.18	-.21	-.27	-.25	.17
11	Free Tapping-slow $_{\text{Variability}}$	-.08	-.08	-.04	-.01	-.01	.06	.11	.14	.13	.1	-	.18	0	.03	.25	-.02	-.11	0	.03	.14	.08	.12	.01	.1	-.15
12	Entrainment-fast $_{\text{Timing error}}$	-.15	-.21	-.23	-.26	.03	.2	.12	-.04	.13	.19	.18	-	.18	.28	.02	-.17	-.11	-.12	-.19	-.2	-.07	-.17	-.13	-.13	-.14
13	Entrainment-fast $_{\text{Variability}}$	-.16	-.11	-.26[a]	-.25	.24	.01	.04	.03	.01	.07	0	.18	-	.08	.14	.04	.06	-.09	-.14	-.13	-.23	-.02	-.16	-.11	-.18
14	Free Tapping-fast $_{\text{Timing error}}$	-.16	-.07	-.12	-.18	-.08	.18	-.14	.22	-.13	.07	.03	.28[a]	.08	-	.05	-.09	-.07	-.1	-.27	-.15	-.08	-.06	-.2	-.16	.05
15	Free Tapping-fast $_{\text{Variability}}$	-.02	-.05	.05	-.06	.33[b]	.01	.03	0	.16	.08	.25	.02	.14	.05	-	-.02	.05	-.21[a]	-.27	-.14	-.28	-.02	-.23	-.1	-.25
16	Dprime $_{\text{Irregular}}$	.11	.28[a]	.06	.2	-.16	-.26	.03	-.34[b]	.06	-.15	-.02	-.17	.04	-.09	-.02	-	.5	.38	.26	.32	.31	.28	.28	.33	.13
17	Dprime $_{\text{Regular}}$	.26[a]	.36[b]	.13	.18	-.14	-.22	.01	-.4[b]	.11	-.28[a]	-.11	-.11	.06	-.07	.05	.5[c]	-	.24	.16	.23	.24	.2	.28[a]	.34[b]	-.02
18	RAN $_{\text{Total}}$	.12	.31[b]	.08	.14	-.2	-.14	-.01	-.17	.23	-.14	0	-.12	-.09	-.1	-.21	.38[b]	.24	-	.37[a]	.25	.44[a]	.22	.28	.14	.35[a]
19	Word reading speed	.36[b]	.27[a]	.25[a]	.3[a]	-.12	-.15	-.02	-.25[a]	.02	-.28[a]	.03	-.19	-.14	-.27[a]	-.27[a]	.26[a]	.16	.37[b]	-	.55[c]	.79[c]	.32[b]	.84[c]	.5[c]	-.01
20	Word reading accuracy	.5[c]	.4[c]	.23	.28[a]	-.07	-.15	-.05	-.34[b]	.04	-.16	.14	-.2	-.13	-.15	-.14	.32[b]	.23	.25[a]	.55[c]	-	.48[c]	.64[c]	.63[c]	.64[c]	.06
21	Nonword reading speed	.32[b]	.27[a]	.21	.25[a]	-.3[a]	-.21	-.15	-.2	-.04	-.18	.08	-.07	-.23	-.08	-.28[a]	.31[a]	.24	.44[c]	.79[c]	.48[c]	-	.38[c]	.77[c]	.53[c]	.02
22	Nonword reading accuracy	.45[c]	.39[c]	.02	.06	-.05	-.23	-.05	-.21	-.05	-.21	.12	-.17	-.02	-.06	-.02	.28[a]	.2	.22	.32[b]	.64[c]	.38[c]	-	.47[c]	.66[c]	.03
23	Text reading speed	.44[c]	.31[b]	.22	.31[a]	-.09	-.13	-.11	-.32[a]	0	-.27[a]	.01	-.13	-.16	-.2	-.23	.28[a]	.28[a]	.28[a]	.84[c]	.63[c]	.77[c]	.47[c]	-	.65[c]	0
24	Text reading accuracy	.5[c]	.25[a]	.21	.18	-.07	-.16	-.12	-.35[b]	-.06	-.25[a]	.1	-.13	-.11	-.16	-.1	.33[b]	.34[b]	.14	.5[c]	.64[c]	.53[c]	.66[c]	.65[c]	-	-.06
25	LoE $_{\text{Italian}}$	.01	.11	.08	.1	-.03	-.18	-.11	-.12	.01	.17	-.15	-.14	-.18	.05	-.25	.13	-.02	.35[b]	-.01	.06	.02	.03	0	-.06	-

Note. Significance levels of the correlations above the diagonal line are corrected for multiple comparisons using False Discovery Rate method.
[a] *p* .05, [b] : *p* < .01, [c] : *p* <.001.
